# Cyclin G2 reverses immunosuppressive tumor microenvironment and potentiates PD-1 blockade in glioma

**DOI:** 10.1186/s13046-021-02078-3

**Published:** 2021-08-27

**Authors:** Sen Li, Chenyang Zhao, Jinlan Gao, Xinbin Zhuang, Shuang Liu, Xuesha Xing, Qi Liu, Chen Chen, Shusen Wang, Yang Luo

**Affiliations:** The Research Center for Medical Genomics, Key Laboratory of Medical Cell Biology, Ministry of Education, School of Life Science, China Medical University, No.77 Puhe Road, Shenyang North New Area, Liaoning Province Shenyang, People’s Republic of China

**Keywords:** Cyclin G2, PD-1, LDHA, Glioma, Immunosuppressive microenvironment

## Abstract

**Background:**

Expression of aberrant cyclin G2 is a key factor contributing to cancer biological processes, including glioma. However, the potential underlying mechanisms of cyclin G2 in the glioma tumor immune microenvironment remain unclear.

**Methods:**

Co-immunoprecipitation (co-IP), *in situ* proximity ligation assay (PLA), and *in vitro* kinase assay were conducted to reveal the underlying mechanism by which cyclin G2 regulates Y10 phosphorylation of LDHA. Further, the biological roles of cyclin G2 in cell proliferation, migration, invasion capacity, apoptosis, glycolysis, and immunomodulation were assessed through *in vitro* and *in vivo* functional experiments. Expressions of cyclin G2 and Foxp3 in glioma specimens was determined by immunohistochemistry.

**Results:**

In this study, we found that cyclin G2 impeded the interaction between LDHA and FGFR1, thereby decreasing Y10 phosphorylation of LDHA through FGFR1 catalysis. Cyclin G2 inhibited proliferation, migration, invasion capacity, and glycolysis and promoted apoptosis glioma cells via suppressing Y10 phosphorylation of LDHA. Moreover, we further verified that cyclin G2 reversed the immunosuppressive to antitumor immune microenvironment through inhibiting lactate production by glioma cells. Besides, cyclin G2 potentiated PD-1 blockade and exerted strong antitumor immunity in the glioma-bearing mice model.

**Conclusions:**

Cyclin G2 acts as a potent tumor suppressor in glioma and enhances responses to immunotherapy. Our findings may be helpful in selecting glioma patients for immunotherapy trials in the future.

**Supplementary Information:**

The online version contains supplementary material available at 10.1186/s13046-021-02078-3.

## Background

Gliomas are the most common primary tumors affecting the adult human central nervous system. The highest-grade form of gliomas, glioblastoma (GBM), accounts for more than 50 % of all diagnosed adult gliomas [1, 2]. Despite surgical [3], chemo-radiotherapeutic [4], and anti-angiogenic treatment [5], the median overall survival for patients receiving the standard of care remains poor at approximately 12–15 months [6]. While immunotherapy, particularly agents targeting programmed cell death protein-1 (PD-1)/ programmed death-ligand-1 (PD-L1), has achieved unprecedented success in malignancies such as melanoma [7] and non-small cell lung cancer [8], its efficacy in GBM remains limited [9].

Dysregulated energy metabolism and immune evasion are interdependent and regarded as the two hallmarks of cancer [10, 11]. Like many other cancer cells, glioma cells tend to metabolize glucose into lactate even in oxygen-rich conditions, known as the Warburg effect [12]. Metabolic reprograming is a pivotal mechanism shaping the tumor microenvironment (TME). Immune responses in the TME depend on the nature and concentration of factors, such as cytokines, reactive oxygen species (ROS), growth factors, and most importantly, diffusible metabolites (i.e., lactate) [13, 14]. Lactate dehydrogenase A (LDHA) promotes the conversion of pyruvate to lactate [15]. Deregulation of LDHA has been reported in several malignancies [16, 17]. Moreover, tyrosine 10 (Y10) phosphorylation of LDHA, which is rampant in diverse cancers, is also correlated with several oncogenic tyrosine kinases, including fibroblast growth factor receptor 1 (FGFR1) [18].

Our previous study revealed that cyclin G2 inhibits aerobic glycolysis in glioma cells [19]. Here, we described how cyclin G2 affects immune cells in TME during glioma progression as potential targets for future therapies. Through a series of *in vivo* and *in vitro* experiments, we found that cyclin G2 inhibits Y10 phosphorylation of LDHA catalyzed by FGFR1, consequently inhibiting glycolysis and reverses the acidic TME. These changes were followed by reduced tumor-infiltrating Tregs and lower levels of TGF-β and IL-10, increased proportions of IFN-γ-producing CD4 + and CD8 + T cells, as well as higher levels of IFN-γ and TNF-α. Moreover, overexpressed cyclin G2 levels potentiated the efficacy of PD-1 blockade in the glioma-bearing mice model. Therefore, the findings may be helpful in glioma therapy, particularly in settings where immune checkpoint blockade alone is not effective.

## Methods

### Cell cultures and treatments

U87, U251 and GL261 cells were cultured in DMEM (Gibco, Carlsbad, CA, USA) supplemented with 10 % (vol/vol) fetal bovine serum (FBS, Biological Industries, Israel) and 1 % (vol/vol) penicillin/streptomycin (Gibco) and passaged every 2–3 days in a humidified 37 °C incubator with 5 % CO_2_. U87 and U251 cells were co-transfected with shLDHA and LDHA WT or LDHA Y10F in the presence or absence of pEGFP-N3-CCNG2 as indicated. For bFGF treatment, U87 and U251 cells were cultured in serum free DMEM media for 24 h and then treated with 10 ng/mL bFGF. After 3 h, the cells were collected and subjected to western blotting. Small interfering RNA (siRNA) used to knock down expression of cyclin G2 (siCCNG2) and scrambled non-targeted siRNA (siNC) were purchased from Genepharma Co., Ltd (Shanghai, China). These siRNAs were transfected into glioma cells at final concentration of 100nM using Dharmafect (Dharmacon, Lafayette, USA) according to the manufacturer’s instructions. Lentiviruses for cyclin G2 overexpression (herein OE) were constructed by GeneChem Co., Ltd (Shanghai, China), and a stable cell line was generated as previously described [19].

### Plasmid construction

Short hairpin RNA (shRNA) targeting the LDHA 3’-UTR were constructed into the pLKO.1 vector and the oligonucleotides used for LDHA-specific shRNA were: forward oligo, 5’-CCGGGATCTGTGATTAAAGCAGTAACTCGAGTTACTGCTTTAATCACAGATCTTTTTG-3’, and reverse oligo, 5’-AATTCAAAAAGATCTGTGATTAAAGCAGTAACTCGAGTTACTGCTTTAATCACAGATC-3’. These were annealed to form a fragment containing an *Age* l and an *Eco R*I restriction site overhang and then cloned into a restriction enzyme-digested plasmid to create pLKO.1-shLDHA. pLKO.1-shNc was used as a negative control. LDHA wild-type and mutant were subcloned into the pcDNA3.1 vector with a 3×Flag tag and pET-28a vector. The Y10F LDHA plasmid is a phospho-defective mutant containing the substitution of tyrosine acid residue at position 10 to phenylalanine and is resistant to pLKO.1-shLDHA. The pEGFP-N3-CCNG2 expression vector was generated as described before [20].

### Cell proliferation assay

Cell proliferation assay was performed using CellTiter 96 AQ_ueous_ One Solution cell proliferation assay kit according to the manufacturer’s instructions (Promega, Madison, WI, USA). Briefly, 1 × 10^3^ cells were seeded in a 96-well plate. At 24 h, 48 h, 72 and 96 h post seeding, 20 µl MTS was added to each well and incubated for 3 h at 37℃. Optical density (OD) values were measured at 495 nm with an ultraviolet spectrophotometer (Thermo Fisher Scientific, Waltham, MA, USA).

### Colony formation assay

Cells were seeded in six-well plates at a density of 300 cells/well, and cultured in DMEM for 15days. The colonies were fixed with methanol and then stained with 1 % crystal violet (Solarbio, Beijing, China).

### Cell migration and invasion assay

Cell migration assay was performed using transwell chambers (Corning, NY, USA) on 24-well plates. 5 × 10^4^ cells/well were suspended in serum-free DMEM in upper chamber. Cell invasion assay was performed using transwell chambers coated with Matrigel (BD Biosciences, Franklin Lakes, NJ, USA) and 8 × 10^4^ cells/well were suspended in serum-free DMEM in upper chamber. Lower compartment was filled with DMEM containing 15 % FBS. After 16 h of incubation in 5 % CO_2_ at 37℃, cells invaded to the lower surface were stained with 1 % crystal violet.

### Apoptosis assay

Cell apoptosis was determined using Annexin V-PE apoptosis detection kit (BD Biosciences) according to the manufacturer’s instructions. Briefly, U87 and U251 cells were stained with Annexin V-PE and 7-AAD in the dark for 15 min at room temperature. After incubation, the cells were washed and processed with a FACSFortessa flow cytometer and analyzed by FlowJo software (TreeStar, Ashland, OR, USA). Apoptosis was determined as the total percentage of Annexin V-positive cells.

### Western blotting and co-immunoprecipitation

Tumor cells and T cells were lysed in RIPA buffer containing protease/phosphatase cocktail (Roche, Basel, Switzerland). Protein concentration was determined using BCA assay. Equal amounts of protein samples were separated by 12 % SDS-PAGE and incubated with the specific antibodies. For co-immunoprecipitation, whole-cell lysates (WCL) of U87 or U251 cells were prepared using lysis buffer and centrifuged. 2 mg proteins were pre-cleared and mixed with 8 µg of anti-FGFR1 antibody or mouse IgG, together with 40 µL of Protein A/G PLUS-Agarose (Santa Cruz Biotechnology, CA, USA) and incubated at 4 °C overnight while rotating. Immunoprecipitates were obtained via centrifugation, washed in lysis buffer, mixed with 2×loading buffer and subjected to western blotting. GAPDH was used as a loading control for western blotting analysis. Bands were detected using an ECL plus kit and quantified using the Image J (National Institutes of Health).

### In vitro kinase assay

Recombinant GST-tagged cyclin G2 and FGFR1, as well as His-tagged LDHA WT and LDHA Y10F proteins, were expressed and purified from *E. coli* BL21 (DE3). The respective proteins were incubated at room temperature for 30 min in FGFR1 kinase buffer containing 10mM Hepes (pH 7.5), 150mM NaCl, 10mM MnCl2, 5mM DTT, 0.01 % Triton X-100, and 200µM ATP. Thereafter, samples were fractionated by 12 % SDS-PAGE and immunoblotted with the corresponding antibodies.

### Protein stability assay

U87 and U251 cells stably expressing cyclin G2 were treated with cycloheximide (CHX, 100µM, MedChemExpress, MonMouth Junction, NJ) for the indicated times. The abundance of LDHA was measured by western blotting and analyzed with Image J.

### Isolation and culture of lymphocytes

To isolate lymphocytes, spleens excised from C57BL/6 mice were homogenized with RPMI 1640 medium and filtered through sterile 70 μm cell strainers and then subjected to density gradient centrifugation using Lymphoprep. T lymphocytes were isolated using EasySep™ Mouse T cell isolation kit (STEMCELL) according to manufacturer’s instructions and cultured in RPMI-1640 medium containing 10 % FBS, 1 % penicillin/streptomycin, 50 U/mL recombinant IL-2 (R&D Systems, Minneapolis, MN), 50 µM β-mercaptoethanol (Sigma-Aldrich), 2 mM L-glutamine (Invitrogen, Carlsbad, CA, USA), 1 mM sodium pyruvate (Sigma-Aldrich), 2 µg/mL anti-CD28 (Invitrogen) and 5ug/mL plate-bound anti-CD3 (Invitrogen).

### Flow cytometry analysis

Single cells were blocked with anti-CD16/32 (BD Biosciences), and stained with the following fluorochrome-conjugated antibodies diluted in PBS containing 2 % FBS. For intracellular staining, samples were fixed, permeabilized using eBioscience Foxp3 fixation/permeabilization kit (Invitrogen). Cytokine staining was performed after 4 h of *in vitro* stimulation in phorbol 12-myristate 13-acetate (PMA) and ionomycin in the presence of GolgiPlug (BD Biosciences). T cells stained with isotype control antibody were used as negative controls. All cells were subjected to flow cytometric analysis on a FACSFortessa (BD Biosciences).

### Measurement of cytokines

To determine of extracellular cytokine concentrations, cell culture supernatants were harvested after 18–20 h of incubation, filtered, and stored at -80 °C. These samples were subjected to enzyme-linked immunosorbent assay (ELISA) using respective kits for TGF-β, IL-10, IFN-γ and TNF-α (Beyotime, Shanghai, China). To measure intratumoral cytokine concentrations, each tumor was mechanically disrupted in 1 ml ice-cold PBS. Samples were centrifuged to collect supernatants. The level of cytokines in the supernatant was quantified using ELISA kits following the manufacturer’s instructions.

### Cytotoxicity assay

The cytotoxic activity of cytotoxic T lymphocyte (CTL) was measured by CytoTox96® Non-Radioactive Cytotoxicity Assay kit (Promega). Splenocytes were harvested from the spleen of OT-I mice and stimulated by incubation with 2nM of SIINFEKL (OVA_257− 264_ peptide) (Sigma-Aldrich) to expand CD8 + OT-I T cells. CTLs were isolated using EasySep™ Mouse CD8 + T cell isolation kit (STEMCELL) for cytotoxicity assay. GL261 cells were pulsed with 1 µM of SIINFEKL at 37℃ for 2 h before cytotoxicity assay. Effector (immune cells) were co-cultured with target (carcinoma cells) at various ratios in RPMI-1640 medium containing 2 % FBS for 6 h at 37℃ in round-bottomed 96-well plates. Specific lysis was determined using a supernatant. The percentage of cytotoxicity was calculated using the formula % cytotoxicity = (experimental-effector spontaneous-target spontaneous) / (target maximum-target spontaneous) *100 %.

### Measurement of lactate production and glucose uptake

Lactate secretion in the culture medium and glucose uptake by cells was detected using a commercial Lactate Assay kit and Glucose Uptake Colorimetric Assay kit (Sigma-Aldrich, Santa Clara, CA, USA) as described previously [19].

### Isolation of tumor-infiltrating lymphocytes

Tumor tissues were excised, minced, and digested with 1 mg/ml collagenase IV and 1 mg/mL DNase I in RPMI-1640 (Gibco) for 1 h at 37 °C. The resulting cell suspensions were filtered through 70-µM filters, pelleted, and resuspended in PBS. Tumor-infiltrating lymphocytes were then isolated by density-gradient centrifugation over Lymphoprep gradient (STEMCELL Technologies, Vancouver, Canada) and then centrifuged at 800×g for 20 min and washed twice, followed by flow cytometry analysis.

### Treg polarization

Naïve CD4 + T cells were stimulated and cultured in RPMI-1640 and GL261 conditioned medium (v/v, 1/1) supplemented with 40ng/mL TGF-β (R&D Systems), 60ng/mL IL-2 for 5 days. The cell suspension was centrifuged, and supernatants were collected for ELISA. Cellular proteins were isolated for western blotting, and a subset of cells was used for flow cytometry analysis.

### Animal experiments

The TALEN-targeted *Ccng2* knockout (KO) mice (*Ccng2*^*−/−*^) of C57BL/6 genetic background were generated by Cyagen Biosciences Co., Ltd (Guangzhou, China) [20]. A xenograft model was established for AZD3965. Briefly, 2 × 10^6^ GL261 cells were subcutaneously inoculated to the right-side flank of age-matched male C57BL/6J wild-type (WT) and *Ccng2*^*−/−*^ mice. On day 10 after implantation, each group was further randomized into two groups to receive AZD3965 (30 mg/kg weight, MedChemExpress) and vehicle (given oral gavage daily). The treatment was conducted for 20 days. On day 35, tumors were excised and prepared for downstream analyses. For the xenograft model for α-PD-1, 6 to 8-week-old C57BL/6 mice were implanted subcutaneously on the right flank with 2 × 10^6^ (in 200 µL of PBS) control or cyclin G2 stably overexpressed GL261 cells. On day 6, 9 and 12 post-tumor implantations, the respective groups of mice were intraperitoneally injected with 200 µg α-PD-1 (clone: RMP1-14, BioXCell) or isotype control prepared in 200 µL of PBS. Animal survival rate was recorded every day, and tumors were measured regularly with calipers. Tumor volumes were calculated using the formula: 0.5 × lengths × width × width. For downstream analyses, three mice per group were sacrificed on day 25. OT-I TCR transgenic mice were obtained from Hangsi Biotechnology Co., Ltd (Hangzhou, China). All animal experiments were performed according to the relevant regulatory standards and were performed in accordance with the Animal Ethics Committee of China Medical University guidelines and approved by China Medical University Animal Ethics Committee.

### In situ proximity ligation assay

The *in situ* proximity ligation assay (PLA) was performed using Duolink In Situ Detection kit (Sigma-Aldrich) as described previously [19]. Each red spot represents a cluster of protein-protein interaction, and distinct red spots were detected using confocal fluorescence microscope. The histogram quantifies the number of red spots per cell (*n* = 50 cells in two technical replicates). The results are representative of three independent experiments.

### Immunohistochemistry

Formalin-fixed, paraffin-embedded glioma specimens were obtained from the First Affiliated Hospital of China Medical University. Written informed consent was obtained from the patients who provided clinical samples and approval from the Research Ethics Committee of China Medical University, Shenyang, China. Immunohistochemistry was performed as described previously [19].

### Public database

The data of glioma samples and the corresponding clinical information were obtained from Chinese Glioma Genome Atlas (CGGA; *n* = 301; http://www.cgga.org.cn).

### Statistical analysis

Data analysis and presentation were performed using GraphPad Prism 9 (San Diego, CA, USA). The means of the two groups were compared using two-tailed, unpaired t-tests. For multiple comparisons, analysis of variance (ANOVA) was used. Data of triplicate samples from three independent experiments are presented as the mean ± s.d. Differences in survival were analyzed with the log-rank test and Kaplan-Meier analysis. *P* < 0.05 was considered statistically significant. ^*^p < 0.05; ^**^p < 0.01; ^***^p < 0.001; ns, not significant.

## Results

### Cyclin G2 reduced LDHA Y10 phosphorylation catalyzed by FGFR1

**Fig. 1:**
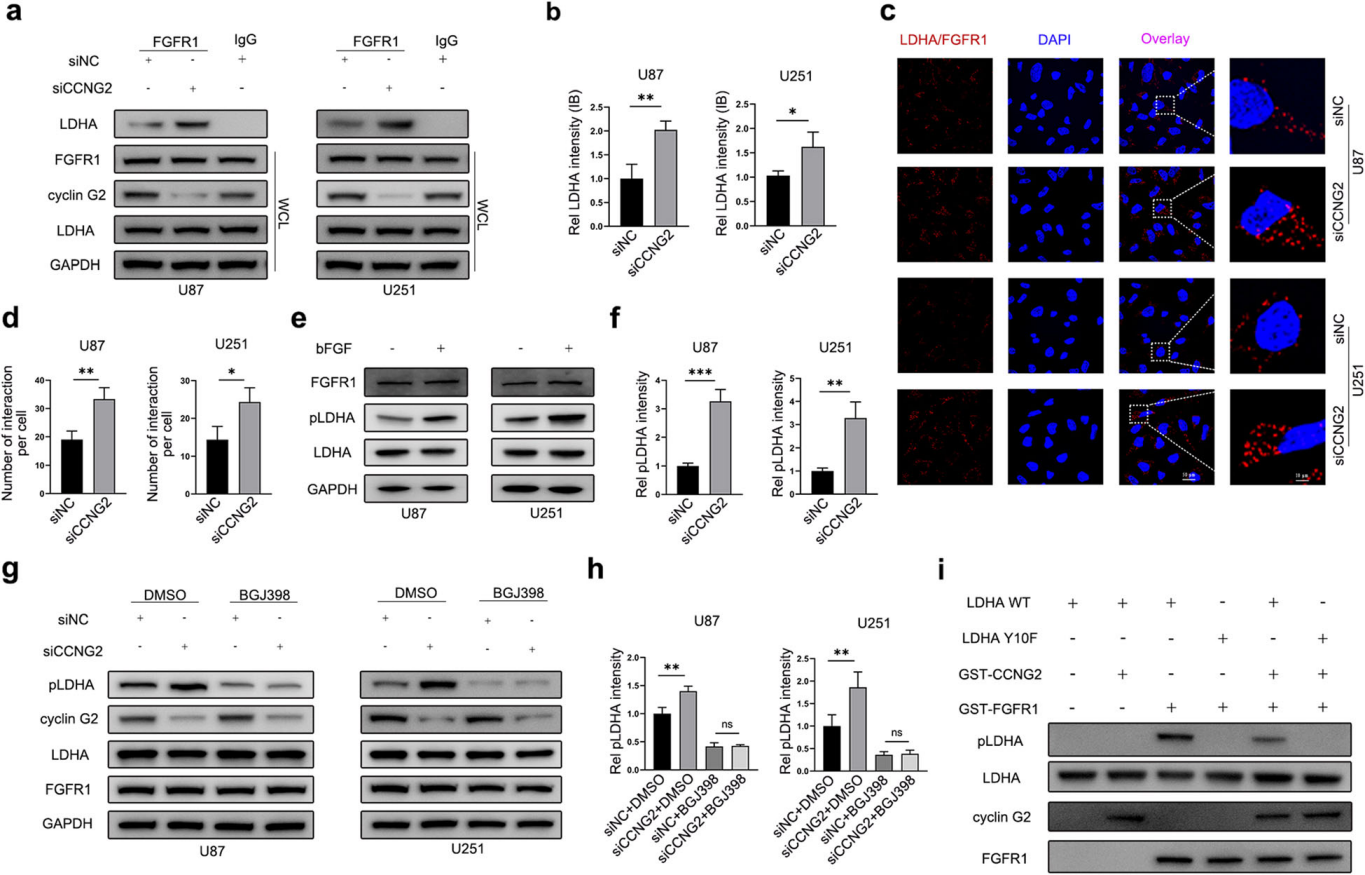
Cyclin G2 reduced Y10 phosphorylation of LDHA catalyzed by FGFR1. **a-b **Western blotting of whole-cell lysates (WCL) from U87 and U251 cells transfected with scrambled non-targeted (siNC) or cyclin G2 siRNA (siCCNG2) using corresponding antibodies, followed by IP with anti-FGFR1, probed with anti-LDHA. Mouse IgG was used as a negative IP control. GAPDH was used as a loading control for western blotting analysis. Bands were quantified using the Image J. **c-d** Proximity ligation assay (PLA) of FGFR1 and LDHA in U87 and U251 cells transfected with siNC or siCCNG2. Red spots indicate FGFR1/LDHA interaction and DAPI was used as a nuclear marker. Original magnification, 1000×. Scale bar, 50 μm. The histogram quantifies the number of red spots per cell (*n* = 50 cells in two technical replicates). The results are representative of three independent experiments. **e-f** After starvation in serum free DMEM media for 24 h, Y10 phosphorylation of LDHA in U87 and U251 cells was detected by western blotting following bFGF stimulation (10 ng/mL, 3 h). **g-h** Western blotting was performed to examine the effect of cyclin G2 on Y10 phosphorylation of LDHA in the presence or absence of BGJ398. **i** I*n vitro* kinase assay and western blotting was performed to examine Y10 phosphorylation of recombinant LDHA WT or LDHA Y10F by FGFR1 in the presence or absence of cyclin G2. Results shown are representative of two independent experiments. Data from three independent experiments are presented as the mean ± s.d. ^*^p < 0.05; ^**^p < 0.01; ^***^p < 0.001; ns, not significant

Given that LDHA Y10 phosphorylation is catalyzed by FGFR1, HER2, and Src [21], to dissect the mechanism underlying the alterations in LDHA Y10 phosphorylation by cyclin G2, we initially examined the interaction between LDHA and upstream kinase affected by cyclin G2 by co-IP. Co-IP verified that LDHA interacted with FGFR1 and that cyclin G2 knockdown heighten the interaction between LDHA and FGFR1 in U87 and U251 cells (Fig. 1a-b). Additionally, we performed *in situ* PLA to examine the interaction between LDHA and FGFR1. As with co-IP, cyclin G2 knockdown enhanced the interaction between LDHA and FGFR1 (Fig. 1c-d). To further assess whether FGFR1 could phosphorylate LDHA in glioma cells, U87 and U251 cells were cultured for 24 h in serum free DMEM media, prior to treatment with bFGF (10 ng/mL, 3 h). Results revealed that treatment with bFGF increased Y10 phosphorylation of LDHA (Fig. 1e-f), indicating that FGFR1 could phosphorylate LDHA in U87 and U251 cells. Moreover, inhibition of FGFR1 by BGJ398 (10nM; MedChemExpress), the inhibitor of the FGFR family, attenuated LDHA Y10 phosphorylation and eliminated the elevation of Y10 phosphorylation mediated by cyclin G2 knockdown (Fig. 1g-h). Notably, cyclin G2 inhibited LDHA Y10 phosphorylation by blocking the interaction between LDHA and FGFR1. Subsequently, we generated phospho-defective LDHA mutant recombinant protein LDHA Y10F and performed an *in vitro* kinase assay to confirm whether the phosphorylation site is regulated by cyclin G2. Results revealed that FGFR1 directly phosphorylated LDHA WT but not LDHA Y10F. Moreover, GST-CCNG2 did not directly dephosphorylate LDHA at Y10. Instead, it inhibited the Y10 phosphorylation of LDHA WT but not LDHA Y10F (Fig. 1i). Studies have revealed that tyrosine LDHA phosphorylation catalyzed by FGFR1 is vital for the stability of LDHA [22]. In our study, protein stability assay demonstrated that cyclin G2 has no impact on the stability of LDHA (Additional file 1, Figure S1a-b). Thus, it is evident that cyclin G2 inhibits Y10 phosphorylation of LDHA via FGFR1 catalysis.

### Reduced Y10 phosphorylation of LDHA is vital for cyclin G2 mediated antitumor effect in glioma

**Fig. 2:**
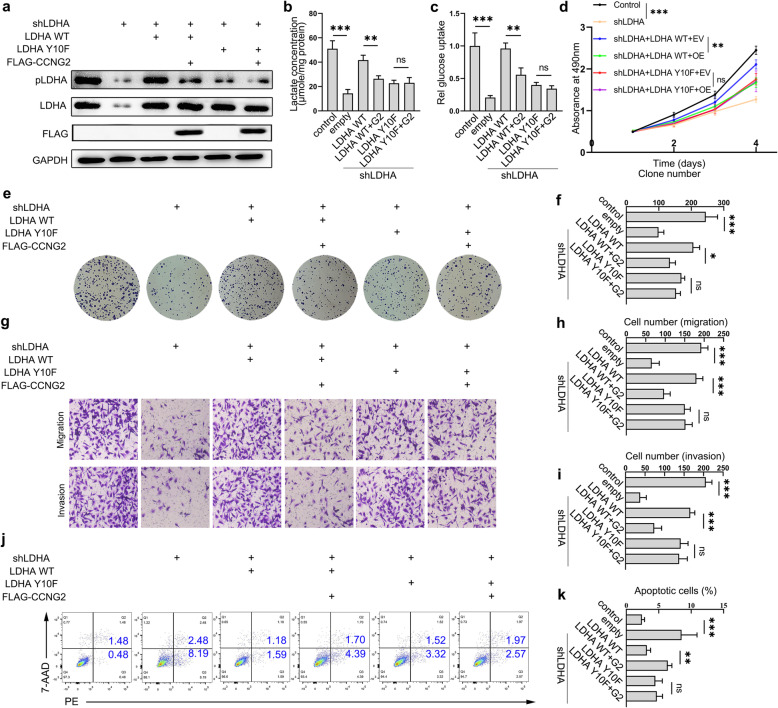
Y10 phosphorylation of LDHA is required for cyclin G2-mediated antitumor functions. **a** Western blotting was performed to examine LDHA expression and Y10 phosphorylation of LDHA in LDHA-knockdown U87 cells with either exogenous LDHA WT or LDHA Y10F expression following cyclin G2 overexpression. **b-c **Lactate production and glucose uptake measured in LDHA-knockdown U87 cells with either exogenous LDHA WT or LDHA Y10F expression following cyclin G2 overexpression. **d** The effect of cyclin G2 overexpression on proliferation of LDHA-knockdown U87 cells with either exogenous LDHA WT or LDHA Y10F expression by MTS. **e-f** The effect of cyclin G2 overexpression on colony formation of LDHA-knockdown U87 cells with either exogenous LDHA WT or LDHA Y10F expression by colony formation assay. **g-i** The effect of cyclin G2 overexpression on migration and invasion capacity of LDHA-knockdown U87 cells with either exogenous LDHA WT or LDHA Y10F expression by cell migration and invasion assay. **j-k** Flow cytometry results showing the influence of cyclin G2 overexpression on apoptosis in LDHA-knockdown U87 cells with either exogenous LDHA WT or LDHA Y10F expression. Data of triplicate samples from three independent experiments are presented as the mean ± s.d. ^*^p < 0.05; ^**^p < 0.01; ^***^p < 0.001; ns, not significant

To assess whether cyclin G2 inhibits proliferation, migration, invasion, glycolysis, or promote apoptosis of glioma cells by modulating Y10 phosphorylation of LDHA, we knocked down endogenous LDHA and restored the expression of wild-type LDHA as well as Y10F mutant LDHA either with or without cyclin G2 overexpression in glioma cells. Results revealed that LDHA knockdown significantly reduces the expression of LDHA, and the level of Y10 phosphorylation of LDHA was strictly restored by LDHA WT, but not LDHA Y10F. Besides, cyclin G2 mediated reduction in LDHA Y10 phosphorylation was diminished in LDHA Y10F mutant versus LDHA WT (Fig. 2a). LDHA knockdown impeded glucose uptake and lactate production, whereas the rescue expression of LDHA WT, but not LDHA Y10F mutant, restored the glucose uptake and lactate production in glioma cells. Reduced glucose uptake and lactate production were mitigated via overexpression of cyclin G2 in LDHA Y10F versus LDHA WT (Fig. 2b-c and Additional file 1 Figure S2a-b). MTS analysis revealed that LDHA knockdown impaired the proliferation, whereas restoring LDHA WT rescued proliferation in glioma. Further, overexpressed levels of cyclin G2 inhibited the proliferation of LDHA WT but not LDHA Y10F (Fig. 2d and Additional file 1 Figure S2c). Similar findings were obtained with colony formation assay and transwell assay (Fig. 2e-i and Additional file 1 Figure S2d-h). In addition, flow cytometry analysis demonstrated that cyclin G2 promoted apoptosis by reducing Y10 phosphorylation of LDHA (Fig. 2j-k and Additional file 1 Figure S2i-j). Conclusively, these findings demonstrated that cyclin G2 suppresses proliferation, migration, invasion, glycolysis and promotes apoptosis of glioma cells by inhibiting Y10 phosphorylation of LDHA.

### Cyclin G2 reversed the immunosuppressive microenvironment in glioma

**Fig. 3:**
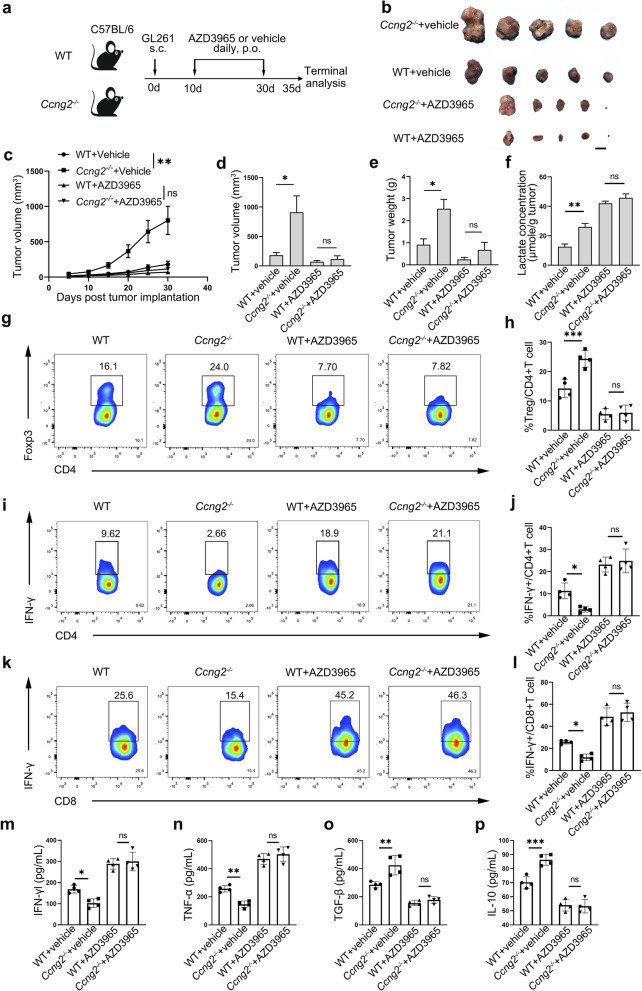
Cyclin G2 knockout resulted in an immunosuppressive microenvironment. **a** Schematic depicting experimental setup. WT and *Ccng2*^*−/−*^ mice were subcutaneously (s.c.) injected with GL261 cells and treated with AZD3965 or vehicle by oral gavage (p.o.). Gross tumors (**b**), tumor growth curves (**c**), tumor volume (**d**) and tumor weight (**e**) at endpoint were measured. *n* = 5. Scale bar, 1 cm. **f** The concentration of lactate in tumors from each group. **g-l** The frequency of Treg, IFN-γ + CD4 + T, IFN-γ + CD8 + T cells in tumors of each group as measured with flow cytometry. *n* = 4 for each group. **m-p** Levels of IFN-γ, TNF-α, TGF-β and IL-10 in tumors of each group determined by ELISA. Data are presented as the mean ± s.d. ^*^p < 0.05; ^**^p < 0.01; ^***^p < 0.001; ns, not significant

The TME, which is composed of tumor cells, stromal cells, immune cells, and the extracellular matrix, is a main platform for the neoplastic process, thereby fostering proliferation, survival, and migration of tumor cells [14, 23]. Tumors can mimic some of the signaling pathways of the immune system to propagate conditions that favor tumor immune tolerance and tumor immunity evasion [24]. Extracellular lactic acid promotes tumor expansion and immune evasion [25, 26]. We hypothesized that cyclin G2 knockout could promote glycolysis, resulting in an immunosuppressive microenvironment and impeded antitumor immunity. Besides, we speculated that blockade of lactate export by AZD3965, an inhibitor of monocarboxylate transporter-1 (MCT1), could eliminate the variation caused by cyclin G2. Thus, we established a syngeneic mouse tumor model by subcutaneously inoculating mouse glioma cell line GL261 in C57BL/6 WT and *Ccng2*^*−/−*^ mice (Fig. 3a). Compared with WT mice, *Ccng2*^*−/−*^ mice displayed a profound acceleration in tumor growth and had a larger tumor volume at the end of the study. The tumor growth curve, tumor volume and tumor weight of *Ccng2*^*−/−*^ and WT mice were comparable when treated with AZD3965 (Fig. 3b-e). Lactate levels were higher in tumors from *Ccng2*^*−/−*^ mice than WT mice, and the elevation was abolished by AZD3965 treatment (Fig. 3f). Subsequently, we investigated immune cell composition in tumors and spleens of each group. Through flow cytometry analysis, we found that *Ccng2*^*−/−*^ mice exhibited an increased frequency of tumor-infiltrating Tregs and decreased frequency of IFN-γ + CD4 + T cells and IFN-γ + CD8 + T cells. However, AZD3965 treatment abrogated the differences in intratumoral immune cell components between *Ccng2*^*−/−*^ and WT mice (Fig. 3g-l). T cell subsets in spleens of *Ccng2*^*−/−*^ and WT mice were comparable (Additional file 1, Figure S3a-c). Next, the secretion of cytokines was examined. Results demonstrated that IFN-γ and TNF-α were lower, and production of immunosuppressive TGF-β and IL-10 were higher in *Ccng2*^*−/−*^ mice versus WT mice treated with vehicle. AZD3965 treatment elevated pro-inflammatory cytokines, reduced immunosuppressive cytokines, and abolished differences between *Ccng2*^*−/−*^ and WT mice (Fig. 3m-p). These results affirmed that tumor-derived lactate can significantly alter the immune profile, while cyclin G2 reverses immunosuppressive TME by inhibiting glycolysis of glioma cells.

### Cyclin G2 mediated immunomodulation via the NF-κB pathway inhibition

**Fig. 4:**
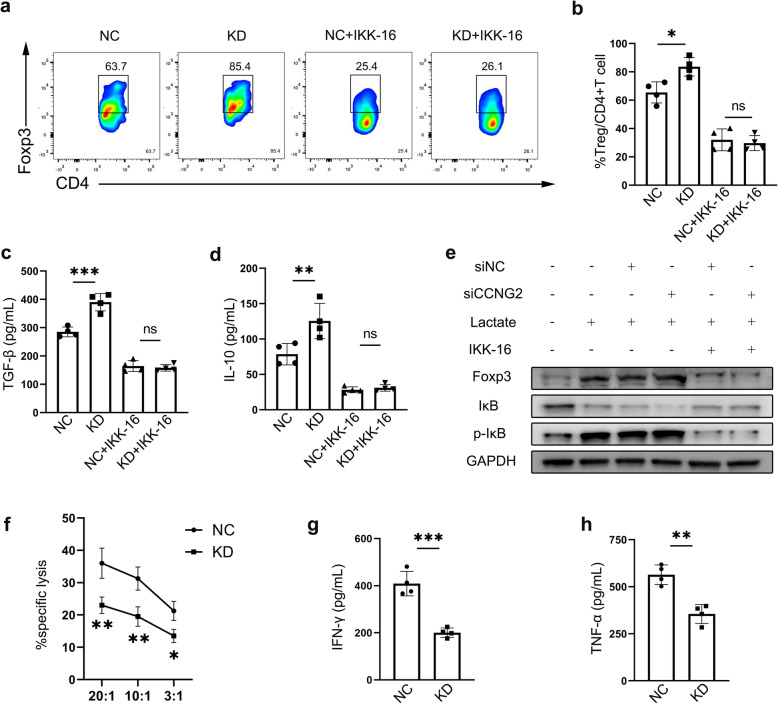
Cyclin G2 mediated immunomodulation via NF-κB signaling. **a-b** Naïve CD4 + T cells were stimulated and cultured in RPMI-1640 and conditioned medium from GL261-NC or GL261-KD for Treg polarization. The frequency of Treg was determined by flow cytometry. **c-d** Levels of TGF-β and IL-10 in the supernatant were determined by ELISA. **e** Expression levels of proteins involved in NF-κB signaling in CD4 + T cells as quantified by western blotting. **f** Results of the cytotoxicity of CTLs as determined by CytoTox96® Non-Radioactive Cytotoxicity Assay. **g-h **Levels of IFN-γ and TNF-α in the supernatant as determined by ELISA. Data of triplicate samples from four independent experiments are presented as the mean ± s.d. ^*^p < 0.05; ^**^p < 0.01; ^***^p < 0.001; ns, not significant

Foxp3, a critical regulatory gene for developing regulatory T cells, reprograms T cell metabolism to function in low-glucose, high-lactate environments [27, 28]. Lactate promotes NF-κB expression, one of the main inducers of Foxp3 transcriptional activation [29, 30]. Therefore, we speculated that cyclin G2 deficiency results in high lactate concentration and may contribute to the suppressive immune microenvironment. To confirm the NF-κB pathway-dependent reduction in the Treg population, we introduced a selective IκBα kinase inhibitor (IKK-16, 2µM; MedChemExpress) in the co-culture system. It turned out that conditioned medium from GL261 cells transfected with siRNA against *Ccng2* (herein GL261-KD) promoted the development of Tregs (Fig. 4a-b) and secretion of TGF-β and IL-10 (Fig. 4c-d). In comparison, the IKK-16 treatment reduced Tregs proportion and cytokines secretion and eliminated the differences between GL261-KD and GL261-NC. Western blotting revealed that lactate increased the phosphorylation of IκB. Also, NF-κB activation resulted in elevated Foxp3 expression, whereas cyclin G2 knockdown increased phosphorylation of IκB and expression of Foxp3. Moreover, we found that blocking IKK activity impeded Foxp3 elevation following cyclin G2 knockdown (Fig. 4e). Tregs induce a dysfunctional state in tumor-infiltrating cytotoxic T lymphocytes (CTLs) resembling T cell exhaustion and are characterized by low expression of effector cytokines and inefficient cytotoxic granule release [31]. To determine the antitumor activity induced by cyclin G2, we made use of well-characterized OT-I transgenic mouse model in which all T cells express the same TCR that is specific for the ovalbumin peptide SIINFEKL (OVA_257 − 264_) [32]. Lymphocytes were isolated from spleens of OT-I mice and co-cultured with conditioned medium from GL261-NC or GL261-KD cells in the presence of SIINFEKL. CTLs were induced and isolated to co-culture with GL261 cells pulsed with SIINFEKL, which can be presented by MHC class I-K^b^ molecules and recognized by OT-I T cells [32]. Results showed lower cytotoxicity of CTLs treated with conditioned medium from GL261-KD compared to GL261-NC (Fig. 4f). Similarly, the conditioned medium from GL261-KD induced CTLs showed lower IFN-γ and TNF-α (Fig. 4g-h), indicating impaired antitumor immunity. Taken together, these findings implicate that cyclin G2 knockdown aggravates lactate-induced activation of the NF-κB pathway, subsequently elevated Foxp3 expression. This results in the promotion of Treg development and impaired cytotoxicity of CTLs.

### Cyclin G2 synergized PD-1 blockade in glioma mice model

**Fig. 5:**
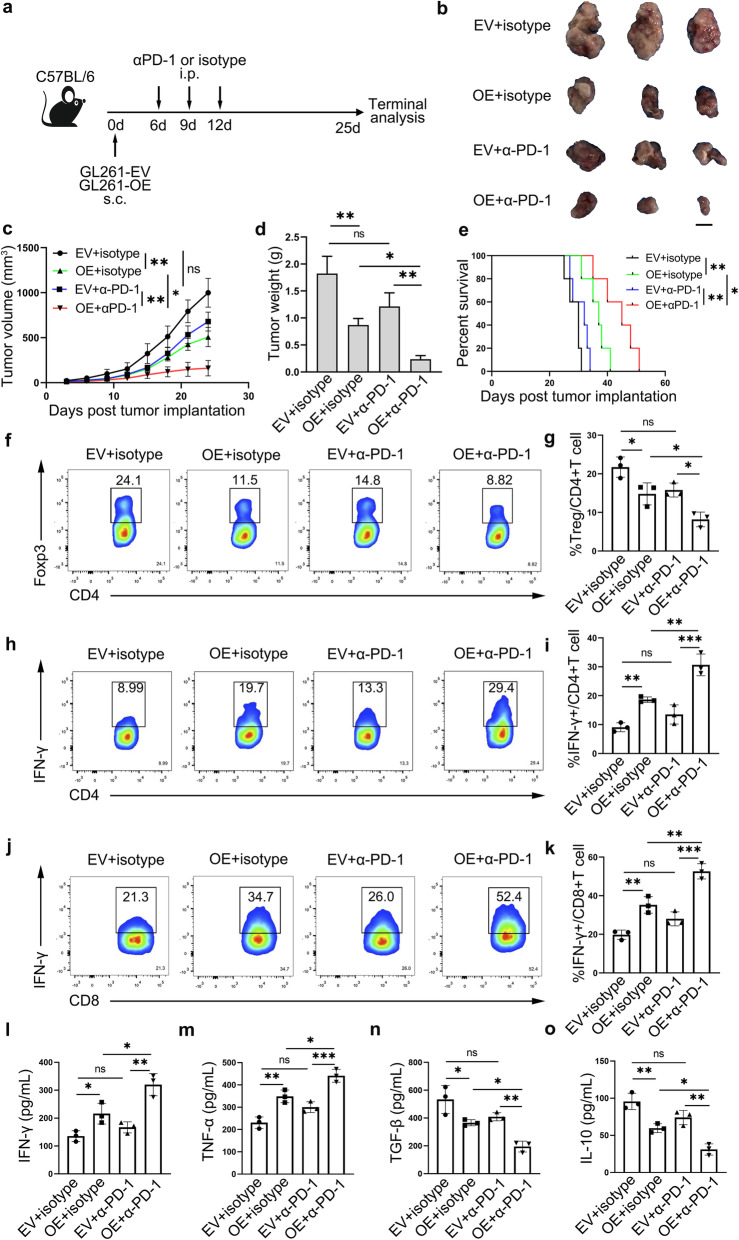
Cyclin G2 potentiated PD-1 blockade in glioma mice model. **a** Schematic depicting experimental setup. C57BL/6 mice were subcutaneously (s.c.) injected with GL261-OE or GL261-EV cells and treated with α-PD-1 or isotype intraperitoneally (i.p.). Gross tumors(**b**), tumor growth curves(**c**), tumor weight(**d**) at the endpoint and overall survival(**e**) are shown. *n* = 5. scale bar, 1 cm. **f-k **Results of flow cytometry showing the frequency of Treg, IFN-γ + CD4 + and IFN-γ + CD8 + T cells in tumors of each group. *n* = 3 for each group. **l-o** Levels of IFN-γ, TNF-α, TGF-β and IL-10 in tumors of each group as determined by ELISA. Data are presented as the mean ± s.d. ^*^p < 0.05, ^**^p < 0.01 and ^***^p < 0.001; ns, not significant

The PD-1/PD-L1 pathway is one of the dominant immune checkpoints in the TME, whereas the PD-1 blockade alone achieved a barely satisfactory outcome in glioma [9]. We hypothesized that cyclin G2 overexpression would potentiate the curative effect of PD-1 blockade owing to a decline in immunosuppressive cells. To evaluate the ability of cyclin G2 to induce tumors to initiate PD-1 blockade and to better mimic the TME in glioma, we used GL261 cells stably expressing cyclin G2 (herein GL261-OE) and empty vector (herein GL261-EV) to inoculate C57BL/6 WT mice (Fig. 5a). Mice harboring GL261-OE showed impeded tumor growth, whereas cyclin G2 overexpression combined with α-PD-1 manifested the smallest tumor volume and lightest weight (Fig. 5b-d). Additionally, the median survival time of mice harboring GL261-OE was longer than mice bearing GL261-EV, which was further prolonged in combination with α-PD-1(Fig. 5e). This affirmed that overexpression of cyclin G2 sensitizes tumors to block PD-1. Notably, overexpression of cyclin G2 combined with α-PD-1 decreased the fractions of Tregs and augmented the frequencies of IFN-γ-producing CD4 + and CD8 + T cells in tumors (Fig. 5f-k). Besides, higher levels of IFN-γ and TNF-α and lower levels of TGF-β and IL-10 were observed in GL261-OE tumors compared with GL261-EV tumors. In mice harboring GL261-OE and treated with α-PD-1, we observed the highest pro-inflammatory cytokines levels and lowest immunosuppressive cytokines levels (Fig. 5L-o). To sum up, overexpression of cyclin G2 augmented immune response and potentiated PD-1 blockade, hindered tumor growth, and prolonged the survival time.

### Cyclin G2 negatively correlates with tumor-infiltrating Tregs in glioma

**Fig. 6:**
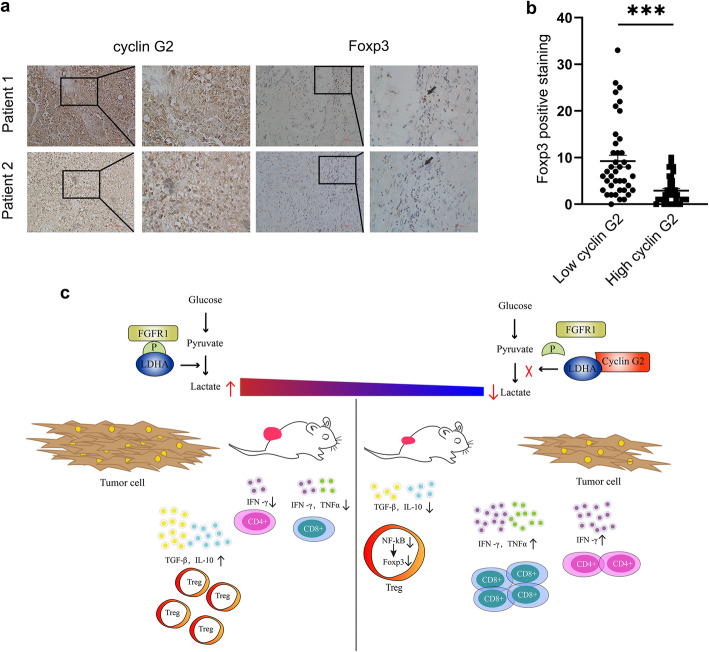
Cyclin G2 negatively correlated with tumor-infiltrating Tregs in glioma. **a** Representative immunohistochemical staining images for cyclin G2 and Foxp3 in glioma specimens. Magnification, 200×, 400×. **b** Foxp3 positive staining in patients with low cyclin G2 expression and high cyclin G2 expression (*n* = 40 per group). Data are presented as the mean ± s.d. ^***^p < 0.001. **c** A schematic model depicting the functions of cyclin G2 in the TME of glioma

Tregs are associated with poor prognosis in several cancers, including glioma [33]. Since initially cyclin G2 was observed to suppress Tregs, we analyzed the CGGA database to explore the correlation between cyclin G2 and Foxp3. Results indicated a negative correlation between cyclin G2 and Foxp3, which uncovers a possible negative impact of cyclin G2 on tumor-infiltrating Tregs in glioma (Additional file 1, Figure S4a). Moreover, overall survival analysis revealed that high expression of cyclin G2 is associated with better overall survival in glioma patients (Additional file 1, Figure S4b). Besides, we subjected glioma specimens to immunohistochemical analysis to validate the negative correlation between cyclin G2 and Foxp3 (representative staining photographs are shown in Fig. 6a). Glioma specimens were divided into two groups based on the expression of cyclin G2. As predicted, Foxp3 staining was significantly higher in the patients with low expression of cyclin G2 than patients with increased expression of cyclin G2 (Fig. 6b). Taken together, these results demonstrate that cyclin G2 negatively correlates with tumor-infiltrating Tregs and plays an essential role in glioma progression.

## Discussion

Cyclin G2 has been implicated as a tumor suppressor in several cancers because it impedes proliferation, migration, invasion, and cycle cell progression of cancer cells [34, 35]. However, the role of cyclin G2 in modulating the immune microenvironment of glioma had not been explored. In the present study, we have revealed a previously uncovered role of cyclin G2 in the tumor immune microenvironment of glioma. Our findings showed that cyclin G2 exerts an anti-immunosuppressive effect by suppressing Y10 phosphorylation of LDHA catalyzed by FGFR1.

We previously demonstrated that cyclin G2 associated with LDHA and inhibited Y10 phosphorylation of LDHA in glioma cells [19], though the underlying mechanism remained elusive. Here, to clarify the mechanisms by which cyclin G2 inhibits LDHA activity and glycolysis, we assessed the upstream kinase of LDHA and found that cyclin G2 inhibited both the interaction between LDHA and FGFR1 and Y10 phosphorylation of LDHA catalyzed by FGFR1. Besides, protein stability assay demonstrated that cyclin G2 has no impact on the stability of LDHA. Knockdown and rescue assay demonstrated that Y10 phosphorylation of LDHA is essential for cyclin G2 mediated antitumor effect. Herein, we revealed that cyclin G2 inhibits the Y10 phosphorylation of LDHA catalyzed by FGFR1, resulting in reduced activity of LDHA.

LDHA executes the final step of glycolysis, and the resultant lactate production leads to a relatively low pH allowing cancer cells to survive immune evasion by diminishing T and NK cell activation [17]. Cytotoxic and effector T cells rely on glycolysis [36], thus become ineffective in the glucose-depleted environment. On the contrary, Tregs primarily rely on oxidative phosphorylation [28], implying that cyclin G2 potentially exerts an antitumor role in glioma by reversing the acidic TME, in consideration of the inhibition of glycolysis by cyclin G2. We, therefore, hypothesized whether cyclin G2 affects immune cell composition during tumor regression in residual tumors. Using the mice model, we found a higher proportion of tumor-infiltrating Tregs in *Ccng2*^*−/−*^ mice compared with WT mice, this was consistent with the intratumoral lactate production. In contrast, tumor-infiltrating IFN-γ-producing CD4 + and CD8 + T cells were reduced in *Ccng2*^*−/−*^ mice. Moreover, pro-inflammatory cytokines were downregulated, whereas immunosuppressive cytokines were upregulated. However, MCT1 inhibition by AZD3965 suppresses lactate exportation, leading to low intracellular pH and feedback inhibition of glycolysis in tumor cells [37], which may not affect immune cells. A recent study revealed that AZD3965 selectively inhibited lactate exportation of tumor cells and augmented cancer immunotherapy [38]. Further, lactate was confirmed to be associated with an immunosuppressive microenvironment. Collectively, cyclin G2 knockout enhances immunosuppressive function by increasing lactate release.

Tregs are highly immune-suppressive subsets of CD4^+^ T cells characterized by the expression of Foxp3, a master regulatory transcription factor that can be regulated by NF-κB, thereby providing a metabolic advantage in a low-glucose and lactate-rich environment [28, 29]. We found that cyclin G2 reduced the frequency and cytokines of Tregs via NF-κB, whereas IKK-16 diminished the reducing frequency and cytokines of Tregs via cyclin G2. Tregs can inhibit cytotoxic CD8 + T cells via various mechanisms [39], and TGF-β can directly suppress CTL immune function [40]. Further, we revealed that cyclin G2 overexpression augments cytotoxicity of CTLs. These findings concur with a previous study that reported lactate impaired IFN-γ in tumor-infiltrating T cells [17].

Beneficial effects of PD-1/PD-L1 blockade have been reported in several cancers [41]. However, checkpoint blockade has revealed limited success as monotherapy for glioblastoma due to immunosuppressive tumor microenvironment [42]. Neutralizing tumor milieu may not only suppress metastatic capacity but could also improve targeting of the tumor by immune effector cells, and this has achieved success in immunotherapy [43]. Conclusively, based on other observations on the influence of lactate on T cells, these results suggest that selective targeting of glycolysis in tumor cells could reverse immunosuppressive TME, restore immune cell activation and immune response against tumor cells. Considering the anti-Warburg effect of cyclin G2, we used overexpressed cyclin G2 combined with α-PD-1 to achieve efficient metabolic therapy and immunotherapy of glioma. For the *in vivo* study, we selected *Ccng2*^−/−^ mice because MEFs from *Ccng2*^−/−^ mice showed a higher level of glycolysis [19]. However, other cell types in the TME, such as tumor and stromal cells, exhibited a higher level of glycolysis, which is a reliable source of lactate [44, 45]. Therefore, since most lactate in the TME was generated from tumor cells, we chose C57BL/6 WT inoculated with GL261 cells stably expressing cyclin G2. Overexpression of cyclin G2 reduced the tumor-infiltrating Tregs and dramatically shifted the balance in favor of IFN-γ + CD4 + and IFN-γ + CD8 + T cells over Tregs in tumors. Combined cyclin G2 overexpression with α-PD-1 treatment exhibited the highest survival rate owing to the efficient synergistic effect on tumor inhibition. Moreover, an elevated proportion of IFN-γ + CD8 + T cells in cyclin G2 overexpression plus α-PD-1 group demonstrated a highly effective cellular immunity activation than in other groups. Overexpression of cyclin G2 generated immune-favorable TME, evident by the reduced proportion of Tregs and elevated tumor-infiltrating IFN-γ + CD4 + and IFN-γ + CD8 + T cells, as well as low levels of immunosuppressive cytokines and elevated cytotoxic cytokines.

Several preclinical and clinical trials suggest that Tregs hamper immune surveillance against cancer in healthy individuals, prevent the development of effective antitumor immunity in patients, and promote tumor progression [46]. Through bioinformatic analysis, low expression of cyclin G2 has been implicated with high expression of Foxp3 and poor prognosis in glioma. Moreover, in this study, we confirmed the negative correlation between cyclin G2 and Foxp3 in glioma specimens.

In conclusion, we revealed that cyclin G2 inhibits the proliferation, migration and invasion, glycolysis of glioma cells and promotes apoptosis by reducing lactate release by tumor cells. This occurs when Y10 phosphorylation of LDHA is inhibited via FGFR1 catalysis. This reduced lactate production by tumor cells reshaped the tumor acidity, causing upregulation of IFN-γ + CD4 + T and IFN-γ + CD8 + T cells and reduction of Tregs, as well as upregulated pro-inflammatory and downregulated pro-tumorigenic cytokine levels (Fig. 6 C). We present the first report on cyclin G2 role in subverting the immunosuppressive microenvironment by inhibiting the Y10 phosphorylation of LDHA catalyzed by FGFR1. Moreover, cyclin G2 overexpression potentiated the therapeutic effect of α-PD-1. Taken together, we concluded that cyclin G2 exerts its tumor suppressor functions by regulating lactate secretion. Furthermore, the expression of cyclin G2 may have prognostic significance and be utilized as a predictive biomarker to select glioma patients for immunotherapy.

## Conclusions

Cyclin G2 acts as a potent tumor suppressor in glioma and enhances responses to immunotherapy. Our findings may be helpful in selecting glioma patients for immunotherapy trials in the future.

## Supplementary Information

**Additional file 1: Supplementary Figure S1.** Cyclin G2 had no effect on the stability of LDHA. (a) U87 and U251 cells were treated with CHX for the indicated times and the level of LDHA was determined by Western blot. (b) Expression level of LDHA relative to that of GAPDH is shown and the slopes of two curves were compared using GraphPad Prism. **Figure S2.** Y10 phosphorylation of LDHA is required for cyclin G2-mediated antitumor functions. (a-b) Lactate production and glucose uptake were mesaured in LDHAknockdown U251 cells with either exogenous LDHA WT or LDHA Y10F expression affected by cyclin G2 overexpression. (c) The effect of cyclin G2 overexpression on proliferation in LDHA-knockdown U251 cells with either exogenous LDHA WT or LDHA Y10F expression as determined by MTS. (d-e) The effect of cyclin G2 overexpression on colony formation of LDHAknockdown U251 cells with either exogenous LDHA WT or LDHA Y10F expression. (f-h) The impact of cyclin G2 overexpression on migration and invasion capacity of LDHA-knockdown U251 cells with either exogenous LDHA WT or LDHA Y10F expression as determined by cell invasion assay. (I-J) The effect of cyclin G2 overexpression on apoptosis of LDHA-knockdown U251 cells with either exogenous LDHA WT or LDHA Y10F expression as determined by flow cytometry. Data are presented as the mean ± s.d. *p < 0.05; **p < 0.01; ns, not significant. **Figure S3.** Cyclin G2 knockout did not affect T cell compositions in spleens. (a-c) Results of flow cytometry analysis showing the frequency of Treg, IFN-γ+CD4+, IFN-γ+CD8+ T cells in spleens of WT and *Ccng2*-/- mice. **Figure S4.** Low cyclin G2 expression was associated with high expression of Foxp3 and poor prognosis. (a) Correlation analysis between cyclin G2 and Foxp3 in CGGA database. (b) Overall survival of patients with high (*n* = 131) or low cyclin G2 (*n* = 131) expression. 

## Data Availability

The dataset(s) supporting the conclusions of this article is(are) included within the article (and its additional file(s)).

## Abbreviations

GBM: Glioblastoma
PD-1: Programmed cell death protein-1
PD-L1: Programmed death-ligand-1
TME: Tumor microenvironment
ROS: Reactive oxygen species
LDHA: Lactate dehydrogenase A
FGFR1: Fibroblast growth factor receptor 1
CTLs: Cytotoxic T lymphocytes
siRNA: Small interfering RNA
shRNA: Short hairpin RNA
WT: Wild-type
