# Supplementary material for: Cyclin G2 reverses immunosuppressive tumor microenvironment and potentiates PD-1 blockade in glioma
Source: J Exp Clin Cancer Res. 2021 Aug 27;40:273. doi: 10.1186/s13046-021-02078-3 (PMC8400712; doi:10.1186/s13046-021-02078-3)
Supplement: Supplementary file 1 — Additional file 1: Supplementary Figure S1. Cyclin G2 had no effect on the stability of LDHA. (a) U87 and U251 cells were treated with CHX for the indicated times and the level of LDHA was determined by Western blot. (b) Expression level of LDHA relative to that of GAPDH is shown and the slopes of two curves were compared using GraphPad Prism. Figure S2. Y10 phosphorylation of LDHA is required for cyclin G2-mediated antitumor functions. (a-b) Lactate production and glucose uptake were mesaured in LDHAknockdown U251 cells with either exogenous LDHA WT or LDHA Y10F expression affected by cyclin G2 overexpression. (c) The effect of cyclin G2 overexpression on proliferation in LDHA-knockdown U251 cells with either exogenous LDHA WT or LDHA Y10F expression as determined by MTS. (d-e) The effect of cyclin G2 overexpression on colony formation of LDHAknockdown U251 cells with either exogenous LDHA WT or LDHA Y10F expression. (f-h) The impact of cyclin G2 overexpression on migration and invasion capacity of LDHA-knockdown U251 cells with either exogenous LDHA WT or LDHA Y10F expression as determined by cell invasion assay. (I-J) The effect of cyclin G2 overexpression on apoptosis of LDHA-knockdown U251 cells with either exogenous LDHA WT or LDHA Y10F expression as determined by flow cytometry. Data are presented as the mean ± s.d. *p < 0.05; **p < 0.01; ns, not significant. Figure S3. Cyclin G2 knockout did not affect T cell compositions in spleens. (a-c) Results of flow cytometry analysis showing the frequency of Treg, IFN-γ+CD4+, IFN-γ+CD8+ T cells in spleens of WT and Ccng2-/- mice. Figure S4. Low cyclin G2 expression was associated with high expression of Foxp3 and poor prognosis. (a) Correlation analysis between cyclin G2 and Foxp3 in CGGA database. (b) Overall survival of patients with high (n = 131) or low cyclin G2 (n = 131) expression. [file 13046_2021_2078_MOESM1_ESM.pdf]

Additional file 1

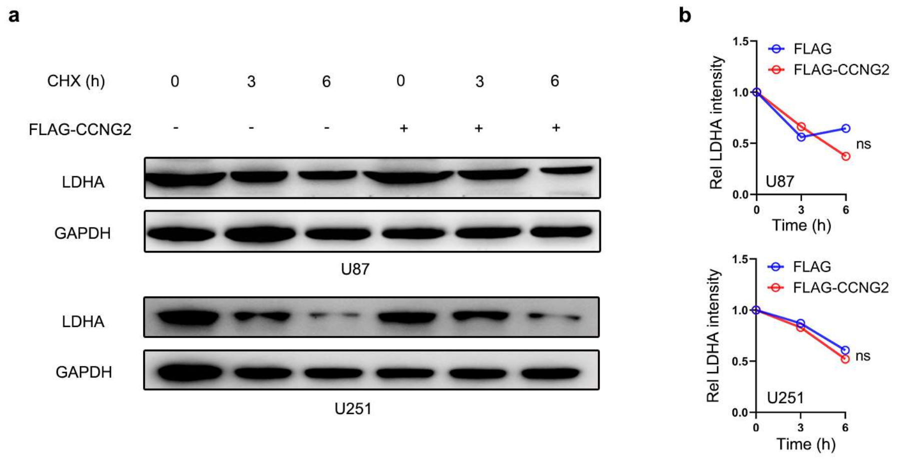

Figure S1. Cyclin G2 had no effect on the stability of LDHA.

(a) U87 and U251 cells were treated with CHX for the indicated times and the level of LDHA was determined by Western blot.

(b) Expression level of LDHA relative to that of GAPDH is shown and the slopes of two curves were compared using GraphPad Prism.

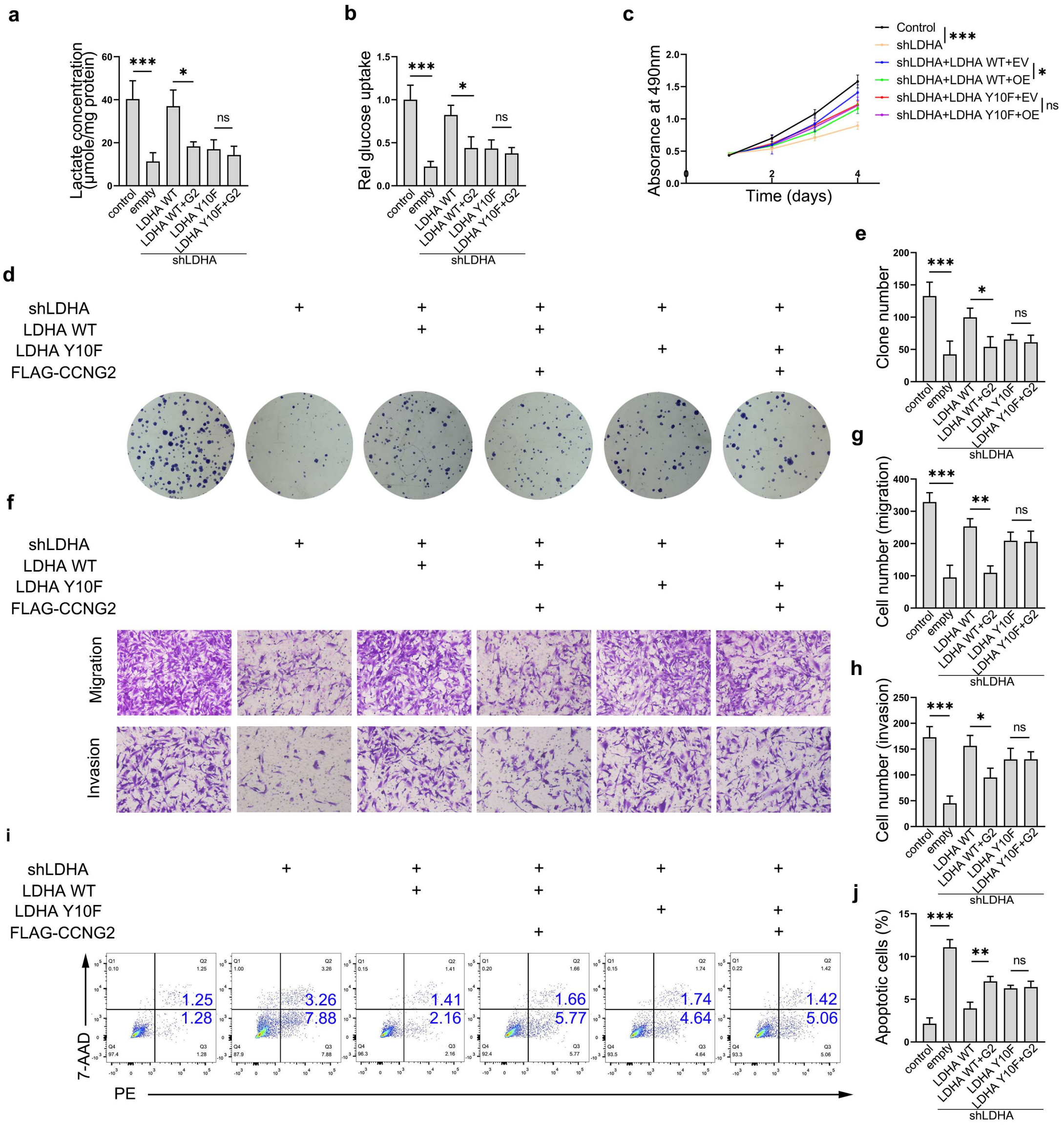

Figure S2: Y10 phosphorylation of LDHA is required for cyclin G2-mediated antitumor functions

(a-b) Lactate production and glucose uptake were mesaured in LDHA-knockdown U251 cells with either exogenous LDHA WT or LDHA Y10F expression affected by cyclin G2 overexpression.

(c) The effect of cyclin G2 overexpression on proliferation in LDHA-knockdown U251 cells with either exogenous LDHA WT or LDHA Y10F expression as determined by MTS.

(d-e) The effect of cyclin G2 overexpression on colony formation of LDHA-knockdown U251 cells with either exogenous LDHA WT or LDHA Y10F expression.

(f-h) The impact of cyclin G2 overexpression on migration and invasion capacity of LDHA-knockdown U251 cells with either exogenous LDHA WT or LDHA Y10F expression as determined by cell invasion assay. (I-J) The effect of cyclin G2 overexpression on apoptosis of LDHA-knockdown U251 cells with either exogenous LDHA WT or LDHA Y10F expression as determined by flow cytometry. Data are presented as the mean ± s.d. *$p < 0.05$; **$p < 0.01$; ns, not significant.

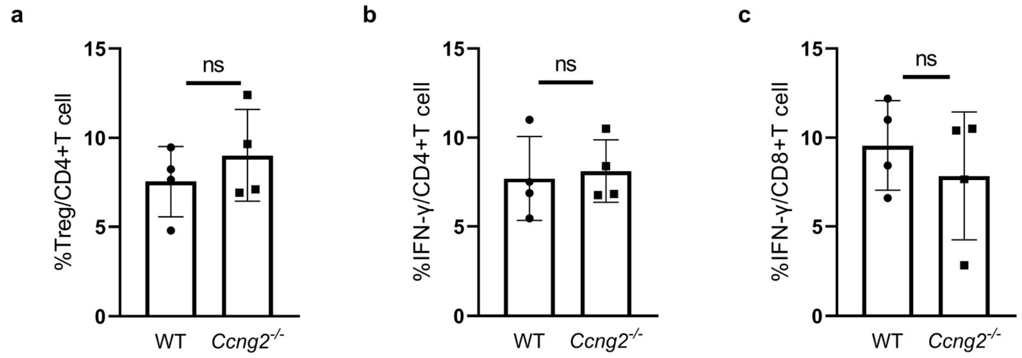

Figure S3: Cyclin G2 knockout did not affect T cell compositions in spleens.

(a-c) Results of flow cytometry analysis showing the frequency of Treg, IFN-γ+CD4+, IFN-γ+CD8+ T cells in spleens of WT and *Ccng2*$^{-/-}$ mice.

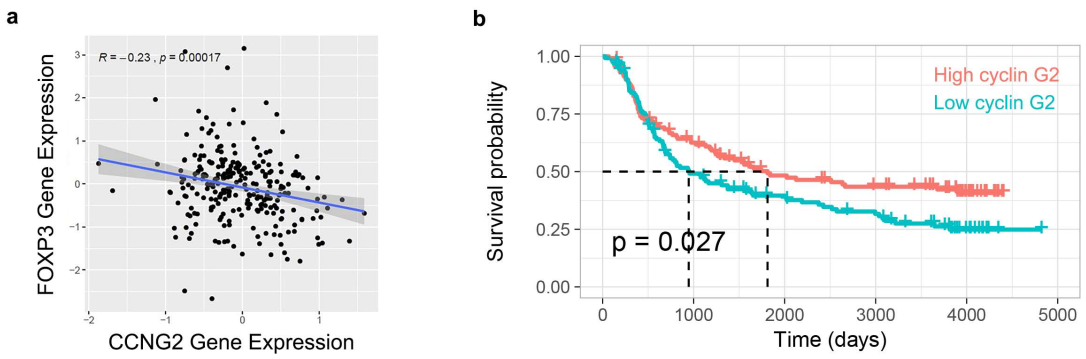

Figure S4: Low cyclin G2 expression was associated with high expression of Foxp3 and poor prognosis.

(a) Correlation analysis between cyclin G2 and Foxp3 in CGGA database.

(b) Overall survival of patients with high (n=131) or low cyclin G2 (n=131) expression.
